# In vivo study of the effects of a portable cold plasma device and vitamin C for skin rejuvenation

**DOI:** 10.1038/s41598-021-01341-z

**Published:** 2021-11-09

**Authors:** Reza Shakouri, Mohammad Reza Khani, Shirin Samsavar, Mahya Aminrayai Jezeh, Fahimeh Abdollahimajd, Seyed Iman Hosseini, Aydin Dilmaghanian, Erfan Ghasemi, Mohammad Reza Alihoseini, Babak Shokri

**Affiliations:** 1 Laser and Plasma Research Institute, Shahid Beheshti University, G.C., P.O. Box 19839-6941, Tehran, Iran; 2 Physics Department of Shahid, Beheshti University, G.C., P.O. Box 19839-6941, Tehran, Iran; 3 Skin Research Center, Shahid Beheshti University of Medical Sciences, Tehran, Iran; 4 Clinical Research Development Unit, Shohada-e Tajrish Hospital, Shahid Beheshti University of Medical Sciences, Tehran, Iran; 5 Faculty of Physics, Shahrood University of Technology, Shahrood, 3619995161 Iran; 6 Department of Basic Sciences, Faculty of Veterinary Medicine, University of Tehran, Tehran, Iran

**Keywords:** Medical research, Physics

## Abstract

Nowadays, cold atmospheric plasma shows interesting results in dermatology. In the present study, a new portable cold plasma was designed for plasma skin rejuvenation (PSR) purposes. This device is safe and easy to use at beauty salons and homes. The effects of this device were investigated on the rat skins. Also, as a new method to improve PSR results, vitamin C ointment was combined with plasma. In this study, there were four groups of 5 Wistar rats. The first group received vitamin C ointment, the second received 5 min of high-voltage plasma, and the third and the fourth groups received 5 min of high- and low-voltage plasma and vitamin C ointment. This process was done every other day (3 sessions per week) for 6 weeks. To evaluate the thermal effect of plasma, the skin temperature was monitored. Also, the presence of reactive species was demonstrated by the use of optical spectroscopy. In addition, mechanical assays were performed to assess the effect of plasma and vitamin C on the tissue’s mechanical strength. The mechanical assays showed a positive impact of plasma on the treated tissue compared to the control group. Also, changes in the collagen level and thickness of the epidermal layer were examined in histological studies. The results indicated an increase in collagen levels after using plasma alone and an accelerated skin reaction after using vitamin C combined with plasma therapy. The epidermal layer’s thickness increased after applying high-voltage plasma, which indicates an increase in skin elasticity. This study demonstrates the positive effect of using the portable plasma device with vitamin C ointment on effective parameters in skin rejuvenation.

## Introduction

Collagen is one of the structural proteins that play an essential role in the body. The presence of this protein in the skin causes the strength, firmness, and elasticity of the skin^1^. Collagen production will decrease from the age of 40 onwards, but in women with aging, there is a 1–2% decrease in collagen production per year after menopause^2^. After the age of 60, this reduction in collagen production will be very significant^3^. This reduction in the production of collagen in the skin causes the skin to lose its elasticity. As a result, an increase in wrinkles, a decrease in smoothness and softness, sagging skin, etc., are observed, and the skin becomes very vulnerable^1,4,5^. Due to the ultraviolet light, diseases such as pigmentation, skin roughness, etc., occur in the skin^6^. Therefore, people are looking for effective and safe solutions to combat these diseases and reach skin rejuvenation. Stimulating fibroblast cells responsible for synthesizing collagen proteins can be a very effective way for skin aging control^7^, which is done in various ways.

Plasma is produced by ionizing neutral gas molecules using energy in the form of electromagnetic fields or heat^8^. In fact, it is an ionized gas consisting of ions, electrons, reactive oxygen species (OH, H_2_O_2_,…), reactive nitrogen species (NO, NO_2_,…), electric field, heat, etc.^9^. Cold plasma has significant potential in the medical field. Particularly in dermatology, it can be used for sterilization or disinfection, treatment of various skin infections, and also for plasma skin rejuvenation (PSR)^10–15^.

Some studies showed that using CAP can improve the underneath tissue’s oxygenation. This may happen because of increasing the temperature (between 30 and 40) and NO that is formed directly or indirectly by CAP. Nowadays, some therapies use oxygenation for skin-rejuvenation. Also, it seems that pressurized oxygen can protect the skin from UVB-induced photoaging in the PSR method^16^. In addition, NO spices have a wide range of biological functions, including neurotransmission, response to immunogens, and smooth muscle relaxation. In addition, some studies showed that NO plays an important role in skin, especially in the proliferation of keratinocytes and fibroblasts^17,18^.

Cold plasma can induce an epidermal thickening probably by keratinocyte proliferation. It can be because of reactive nitrogen spices and reactive oxygen spices on the different cytokines and subsequent cell proliferation in the treated areas^18,19^. Also, Suschek et al. demonstrated that plasma could improve dermal microcirculation because of the vasodilator effect of NO without specific side effects. So, an influx of inflammatory cells and subsequent production of different growth factors and cytokines and stimulation of cell proliferation, including fibroblasts, can be seen because of the increased dermal blood flow^18–21^.

Generally, the plasma hits the skin area after leaving the device and causes sudden warming of the area due to heat transfer to the skin by the excited gas. The activity of fibroblasts increases during dermal regeneration, and by maintaining necrotic epidermis, we have a biological dressing that can promote and decrease the period of recovery^18^. Initial studies have shown that PSR can effectively treat facial skin and non-facial areas with minimal complications.

Comparison between the PSR method and a carbon dioxide laser on an animal specimen shows that all treatment areas regenerated epidermis after 7 days in the PSR-treated skin. An active cellular response below the dermis and epidermis junction is also noticeable. Therefore, PSR can be an excellent alternative to CO_2_ lasers with the appropriate deformation to the skin structure^22^.

Also, using a PSR device to improve the facial skin showed the production of a new collagen band at the junction of the dermis and epidermis with low-density elastin in the upper layers of the dermis 3 months after treatment. There were no signs of ulceration or hypopigmentation. Skin erythema was ultimately improved on average 6 days after treatment^12^.

In addition, PSR was used to treat the skin of hands, neck, and chest with photo damaging. Clinical evaluation of the skin, pigmentation, wrinkle severity, and side effects were performed on different days after treatment. The results indicated significant clinical improvement in treatment areas, considerable reduction of wrinkles and hyperpigmentation, and increased skin softness. The key point is that PSR was associated with moderate improvement in sun-induced skin changes in the neck, chest, and back of the hands with minimal side effects^23^.

According to the studies in this field, PSR seems to be an effective and safe method with minimal side effects^14,24^, and it can be used with other methods; for example, the use of PSR along with cosmetic surgery plays a significant role in promoting healing in the skin of the forehead, eye area, mouth area and middle half of the face^25^. Moreover, This method has shown good potential in wound healing with no hypopigmentation and wound effect^26^.

According to this information, plasma can be considered a suitable method for skin rejuvenation due to its high safety and effectiveness. One of the weaknesses of most PSR devices mentioned in this paper is the need for complicated and costly devices, various, expensive, and complicated equipment. Therefore, the impossibility of using this device at home and in beauty salons. In this research, a portable PSR device for rejuvenation is designed with a low price and is easy to carry and use so that people can use it at home or in the nearest beauty salons without wasting their time and energy. After that, the effects of the portable PSR device on an animal model were studied. Also, as a new method, the possibility of improving the effects of the PSR method by applying vitamin C ointment was investigated, and the mechanical and histological parameters were measured and evaluated. Finally, the results showed that vitamin C ointment could improve the results of the PSR device.

## Results

### Characterization of cold plasma device

**Figure 1:**
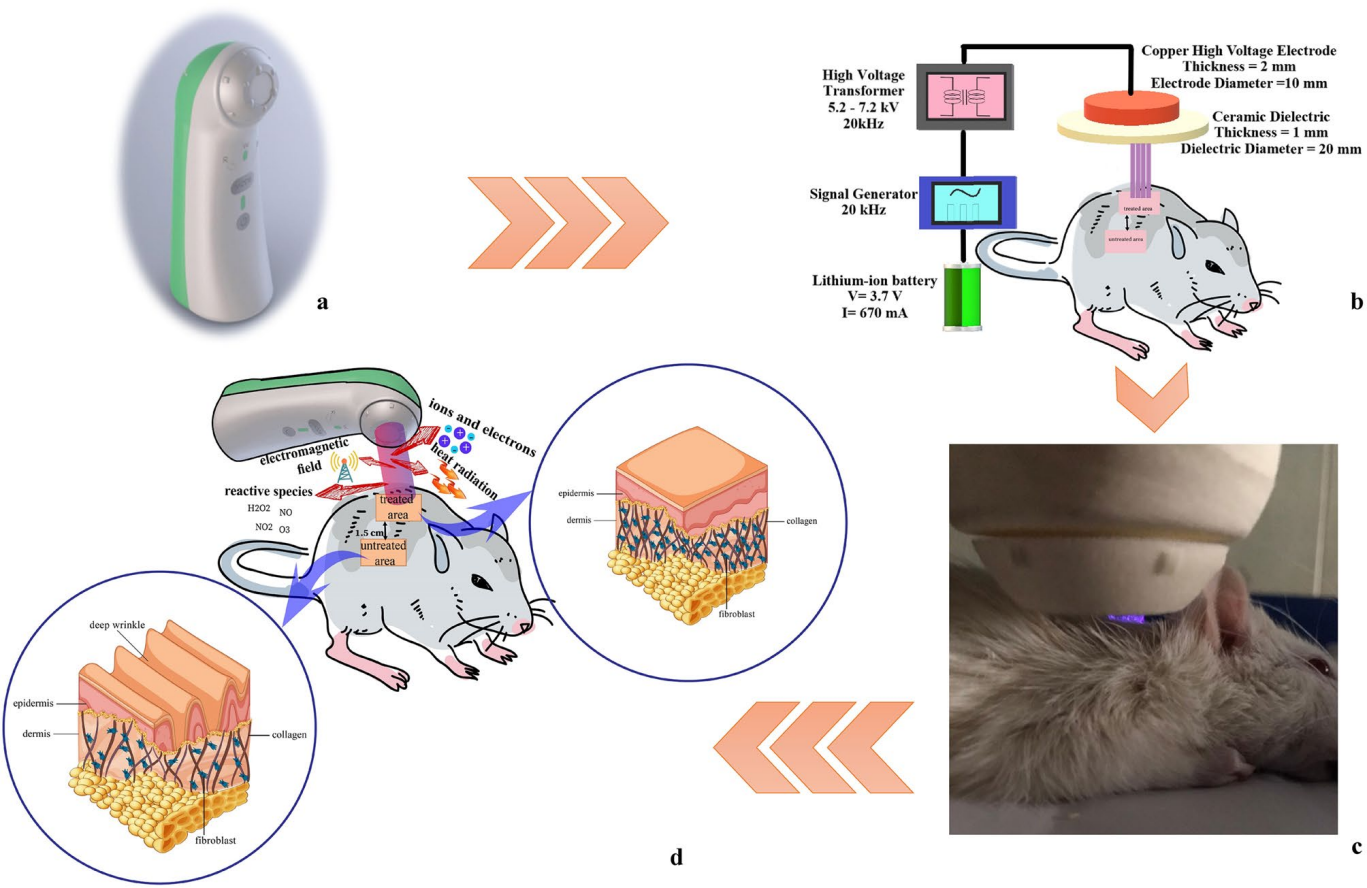
Plasma device and the process of treatment (**a**) The appearance of plasma device, (**b**) the schematic of the internal structure of the plasma device, (**c**) treating skin with cold plasma radiation, (**d**) schematic diagram of treating the skin of a rat by portable plasma device.

**Figure 2:**
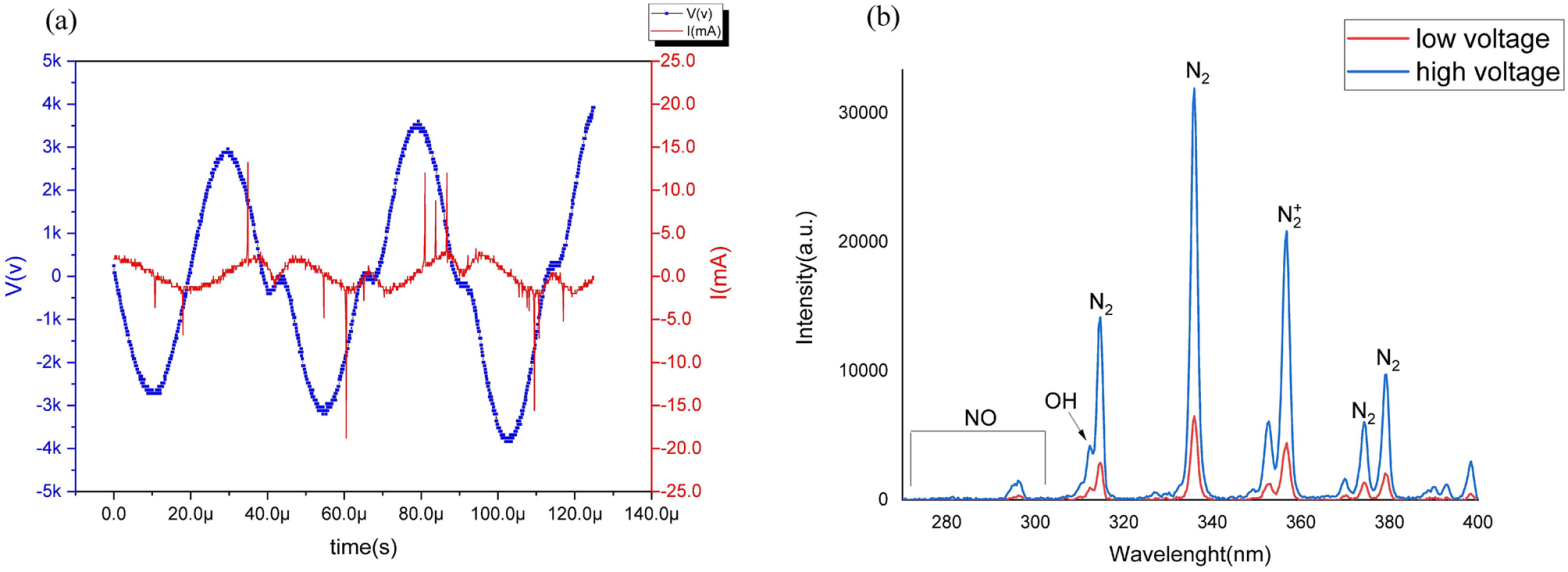
Characterization of cold plasma device (**a**) the voltage (blue line) and current (red line) of plasma device for the high-voltage mode, (**b**) optical emission spectra of the plasma device and excited species produced by it for both high- and low-voltage modes.

Figure 1a,b demonstrate the appearance and schematic structure of the portable cold plasma device. Treating skin with cold plasma radiation and schematic diagram of treating the rat's skin by portable plasma device are presented in Fig. 1c,d. The electrical characteristics for the cold plasma device were measured, and the peak-to-peak voltages and current were 5.2 and 7.2 kV for low and high voltages, respectively. Also, the device’s frequency was 20 kHz for both modes (Fig. 2a). The optical emission spectroscopy of the plasma device at peak-to-peak voltages is 5.2 and 7.2 kV for low- and high-voltage plasma, respectively, which demonstrates the emission of species such as OH (309 nm), NO (297 nm), N_2_/N_2_^+^ (315,337, 358, 375.4 and 380 nm) (Fig. 2b)^2,4,5^.

### Temperature measurements

**Figure 3:**
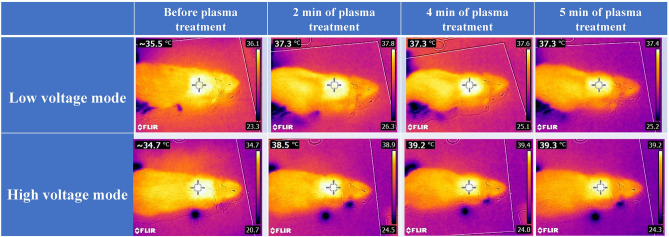
Skin temperatures of rats were measured by an infrared camera, before treatment and after 2, 4, and 5 min of treatment by low- and high-voltage modes of the plasma device.

A thermal camera monitored rats’ skin temperature before and during low- and high-voltage plasma treatments (2,4 and 5 min of plasma treatment). The skin temperature was below 40 °C throughout the therapy (Fig. 3).

### Visual observation of plasma performance

**Figure 4:**
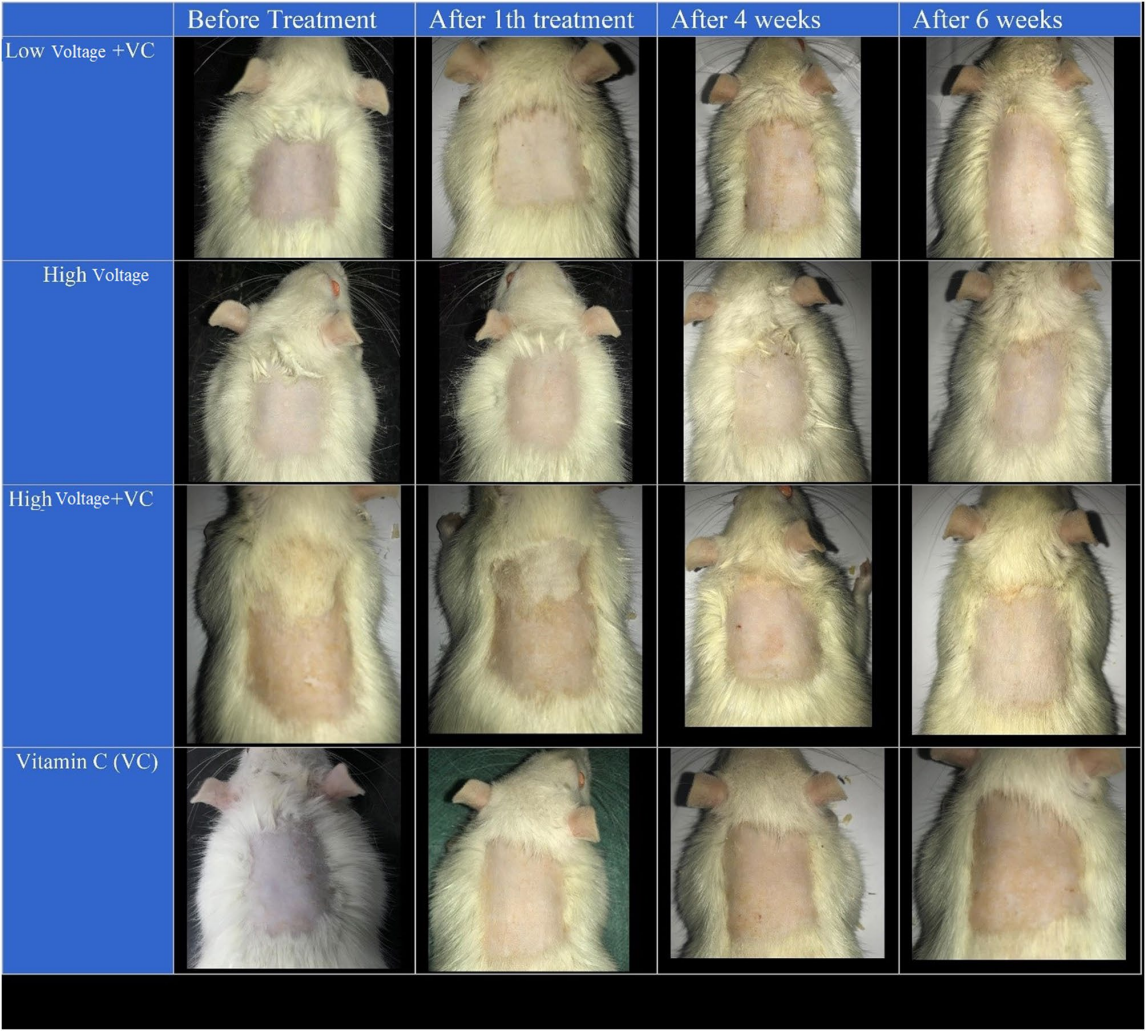
Visual observation of plasma performance in the rats during 6 weeks.

Figure 4 shows the changes in the plasma process up to the sixth week. The first is related to low-voltage plasma processing with vitamin C. The second row is for high-voltage plasma, and the third is for high-voltage plasma with vitamin C. In the bottom row, samples processed with vitamin C alone are displayed. As it turns out, the images do not show significant changes in the skin. The plasma effect on animal specimens is not as good as observed on human samples^18^. Therefore, it was decided to use quantitative experiments to evaluate the effectiveness. However, the images show that a small amount of inflammation develops immediately after processing on the skin's surface. Also, in appearance, the amount of increase in skin whiteness is visible. It is worth noting that observing small color spots on the skin is caused by shortening the hair on its surface. Due to the design of this device as a portable homemade model, the intensity and energy of the plasma are designed in such a way that it does not have destructive and severe effects on the skin due to its use.

### The effects of portable plasma device on mechanical parameters

#### Maximum stress for sample (N/mm^2^)

**Figure 5:**
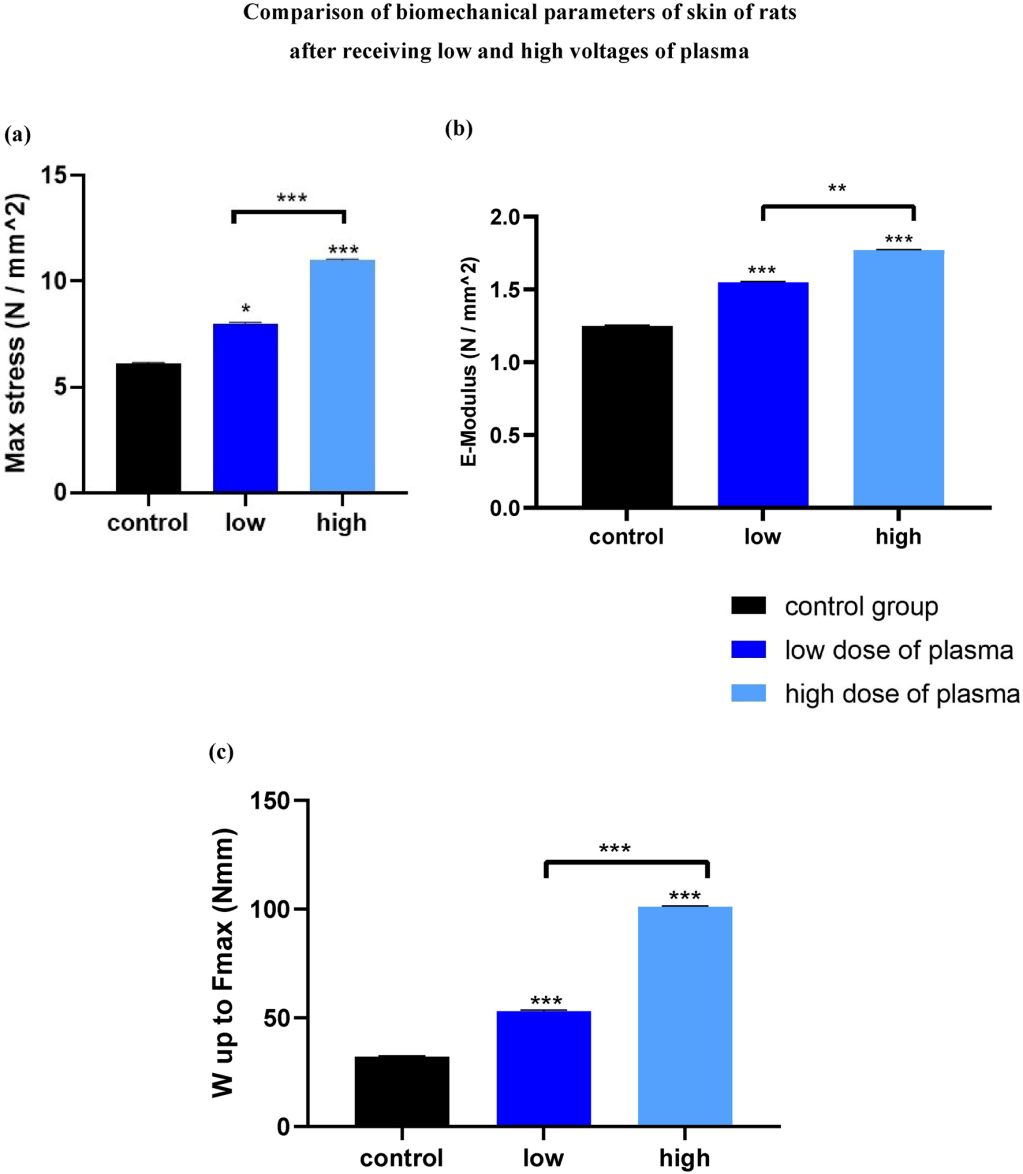
The comparison of biomechanical parameters of rat skins after receiving low and high voltages of plasma (**a**) maximum stress, (**b**) elastic stiffness, (**c**) work up to maximum force [*p < 0.05, **p < 0.01, ***p < 0.001, significant differences compared with the control group (without treatment skin)].

As shown in Fig. 5a, the maximum stress increased with increasing voltage, and the higher-voltage processed sample was able to withstand more force before rupture. This increase is linear and indicates the tolerance of the skin.

#### Elastic stiffness [E-Modulus (N/mm^2^)]

An elasticity module (E-Modulus) is a parameter measuring the resistance of an object or material that is flexibly (indirectly) deformed when stress is applied to it. As can be seen, the specimens’ stiffness and elasticity increased with increasing plasma voltage, and the processed specimens had more stiffness before rupture (Fig. 5b). This increase is relatively linear and indicates the freshness of the skin.

#### Work up to maximum force (Nmm)

As can be seen, the amount of work up to maximum force performed by the device increased with increasing the processed voltage, and the higher-voltage sample required more work for rupture, indicating skin endurance (Fig. 5c).

### The effects of portable plasma device on histological parameters

Collagen plays a key role in the structure of the skin that can cause rejuvenation^1,4,5^. Some of these roles are explained in the discussion section. So, investigating the average thickness of collagen can be a piece of good evidence for the effectiveness of the portable plasma device for rejuvenation.

#### Average thickness of collagen

**Figure 6:**
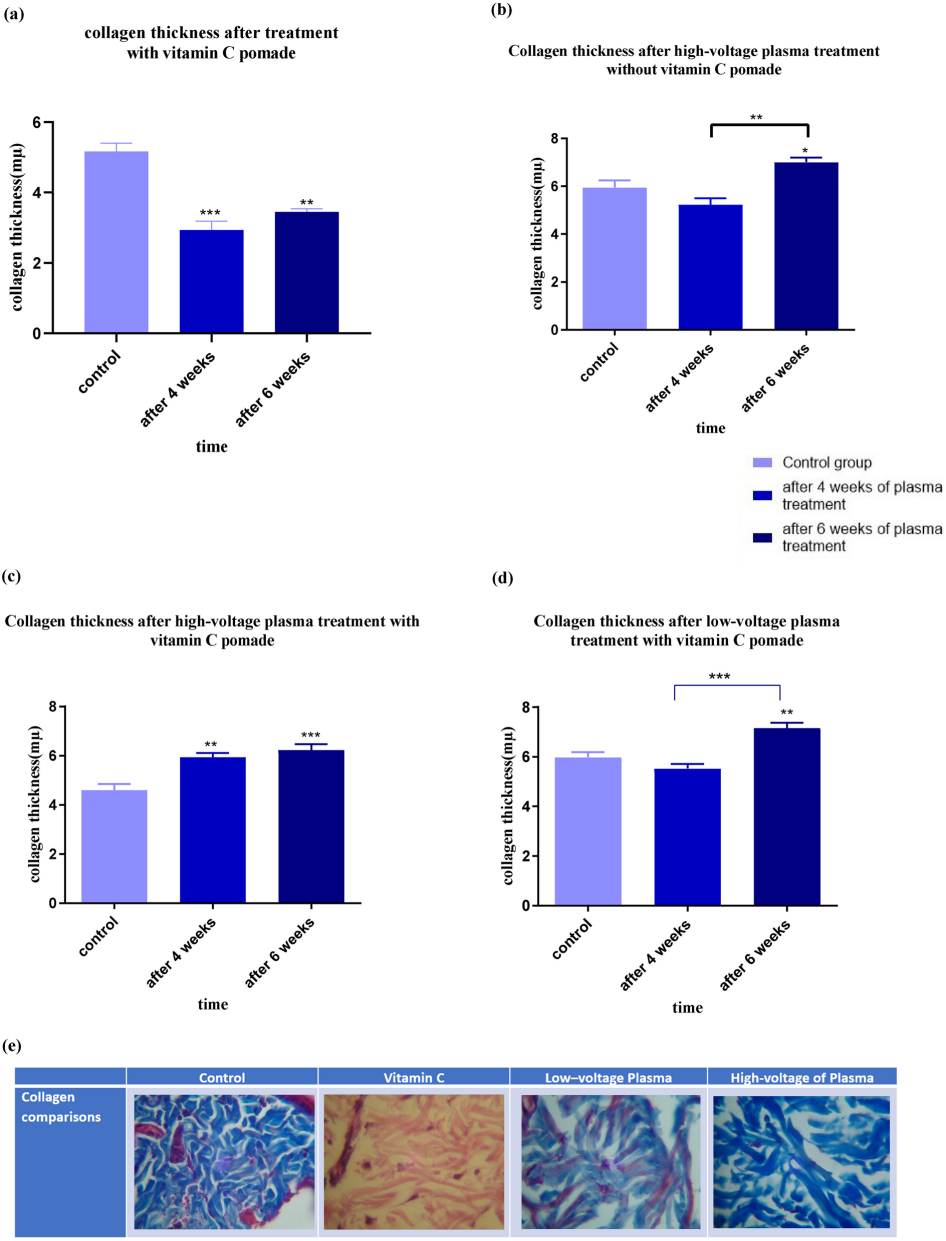
Collagen thickness after 4 and 6 weeks of (**a**) treatment with vitamin C, (**b**) high-voltage plasma treatment without vitamin C pomade, (**c**) high-voltage plasma treatment with vitamin C pomade, (**d**) low-voltage plasma treatment with vitamin C pomade, (**e**) the histopathological images of collagen layer of the treated areas (vitamin C, low voltage plasma and high voltage plasma) compared to the controls (significant differences compared with the control group (without treatment skin), *p < 0.05).

Figure 6 illustrates the changes in collagen levels. Vitamin C treatment after 4 weeks led to a decrease in collagen levels, but there is a slight increase in collagen levels in the sixth week compared to the fourth week; however, its amount is still lower than the control sample (Fig. 6a). As Fig. 6b shows, the collagen level after 4 weeks decreases and then (after 6 weeks) increases, and eventually, the collagen level is higher than the initial value. An increase in collagen levels indicates that the skin responded well to a high-voltage plasma without vitamin C ointment on the last day.

Also, as Fig. 6c shows, the simultaneous use of high plasma and vitamin C voltages has led to an upward trend. However, the previous process, first decreasing, then increasing, has not taken place. This can be the effect of both methods, which causes this process to occur before the first punch, and the skin’s reaction to this method is faster than using a high voltage of plasma alone.

Low-voltage administration of the device with vitamin C showed the first trend again, first decreased and then increased, and eventually, the level of collagen reached a higher level (Fig. 6d). This difference between the collagen level in the control sample and the treated one after 6 weeks of low-voltage administration of the device was lower than the high-voltage administration of the portable plasma with vitamin C ointment, which was expected due to the lower voltage and its fewer active species.

Also, as Golberg et al. claimed in their study, one of the parameters that can be assessed in skin rejuvenation is the proliferation of the epidermis. In addition, they said that epidermal stem cells activation is involved in the thickening/regeneration process^27^. Therefore, the epidermal thickness is investigated in this study as well.

#### Epidermal thickness

**Figure 7:**
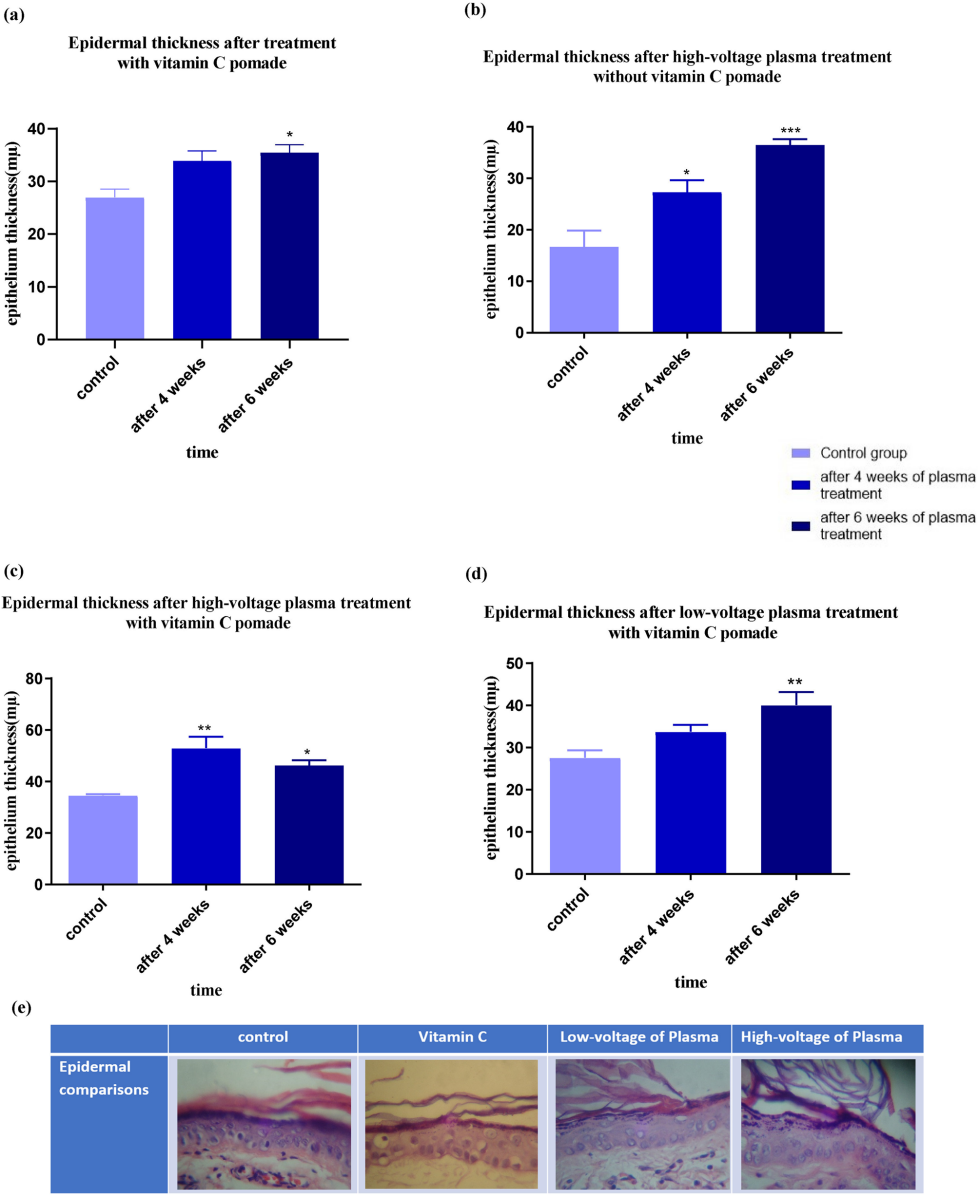
Epidermal thickness after 4 and 6 weeks of (**a**) treatment with vitamin C, (**b**) high-voltage plasma treatment without vitamin C pomade, (**c**) high-voltage plasma treatment with vitamin C pomade, (**d**) low-voltage plasma treatment with vitamin C pomade, (**e**) the histopathological images of the epidermis layer of the treated areas (vitamin C, low voltage plasma and the high voltage plasma) compared to the controls (significant differences compared with the control group (without treatment skin), *p < 0.05, **p < 0.01).

The epidermis layer thickness was examined in all four groups (Fig. 7). Examination of the thickening of the epidermis layer after vitamin C treatment showed an increasing trend (Fig. 7a). Also, high voltage plasma treatment without vitamin C ointment indicates a very upward trend indicating increased skin elasticity (Fig. 7b). Applying a high voltage of plasma with vitamin C showed that the process of changing the thickness of the epidermis layer was initially ascending and then descending, but still higher than the control sample (Fig. 7c). The low-voltage plasma and vitamin C also increased the epidermis layer’s thickness (Fig. 7d). As can be seen, the thickening of the epidermis at all groups corresponded to the mechanical test results. The epidermis layer’s increased thickness also indicates an increase in skin elasticity, observed in mechanical test results.

## Discussion

Plasma warms the skin rapidly after leaving the device and being transferred to the treatment area due to the gas heat transfer to the skin. Heat shocks can cause an upregulation of procollagen type I and procollagen type II. So, they can stimulate the cells to produce more collagen^28,29^. Unlike ablative technologies (ablative lasers), there is no evaporation in the epidermis and burns during treatment in PSR, which is one of the advantages of this device that attracts attention^30^. Plasma is not dependent on interacting with a chromophore. So, it is more uniform than ablative lasers^30^. Fibroblast activity increases during dermal layer rejuvenation^30^. PSR works effectively and with minimal side effects and safely minimizes the risk of unpredictable hot spots and scar^18^. The lines that sometimes remain after laser treatment on the skin around the eyes and mouth have not been seen in this method^30^. Studies on PSR in patients using plasma show continued collagen production, reduced sun elastosis, and increased skin rejuvenation over 1 year after treatment. This method has also been used to treat seborrheic keratosis, actinic keratosis, and viral papillomata. This device is an ideal example for treating the upper and lower eyelids’ skin and wider areas around the eyes^30^.

Each plasma parameter that reacts with skin tissue has its own biochemical, biophysical, and biological effects. The active species NO and heat shock has a more prominent role in skin rejuvenation^9,28,29,31^.

NO is produced in vivo through several types of cells, including skin cells. This molecule is described as a short-lived signaling molecule since it acts as a messenger in cellular communication. This molecule plays an essential role in the nervous, vascular, and immune systems of the human body, for example, in relaxing smooth muscles, controlling blood flow to specific blood vessels in the heart, defensive mechanisms to fight bacteria, parasites and fungi, in vasodilation and nerve transmission, etc.^20,32^. It is also essential to regulate the growth and differentiation of keratinocytes^9^, which are the epidermis’ main constituents and play a defensive role against disease^20^.

Disorder in wound healing or skin tumor formation is associated with an imbalance in the biological synthesis of NO^20,33–36^. Access to exogenous NO production can be important. Non-thermal plasma sources can be a potential tool for medical use to treat these disorders^37,38^.

Exogenous NO, produced through the reaction of plasma with air, has positive effects on the healing of skin wounds due to the increase in bio NO available to tissues present in wound areas^39–42^. Exogenous NO also shortens the healing process of diabetic wounds^43^. Further studies show that exogenous NO normalizes microcirculation and thus reduces inflammation and increases bacterial phagocytosis and fibroblast proliferation. As a result, due to the increase in fibroblast cells, collagen protein synthesis is increased, which leads to repair and thus rejuvenation^31,44^.

Increasing skin temperature to 37–38.5 °C is a stimulant to increase skin keratinocytes’ proliferation and help regenerate tissue^45,46^. In general, tissue temperature should not exceed 40 °C. Temperatures between 40–50 °C can lead to local hyperthermia with cell membranes and molecular structure destruction. It can also lead to edema and cell necrosis. Temperatures above 50 °C lead to denaturation of proteins and collagens and evaporation of cellular liquid^46,47^.

The thermal effect of plasma on the skin leads to heat shock. Reaction to this heat shock stimulates fibroblast cells in the dermis, stimulating heat shock proteins and collagen synthesis through these cells^28^.

To investigate the effect of heat shock, Susan Damz and colleagues cultured human fibroblast cells and exposed them to 45 °C and 60 °C for 2 s. This study showed that with the induced heat shock, the synthesis of type 1 collagen increased. In the next step, they performed a study with 45 °C and 60 °C temperatures and a 16, 10, 8, 4, 2 s processing time. The results showed that at a temperature of 45 °C and 8 to 10 s, the highest synthesis of type 1 collagen occurs^28^.

Several types of research have indicated that vitamin C levels will decrease in aged or photodamaged skin^48–51^; its cause or effect is unknown. Also, it was reported that excessive exposure to oxidant stress via pollutants or UV irradiation is associated with depleted vitamin C levels in the epidermal layer^52,53^. Vitamin C acts as a co-factor for the proline and lysine hydroxylases that stabilize the collagen molecule tertiary structure and promote collagen gene expression^54–62^. Collagen formation is done mainly by the fibroblasts in the dermis, cause the generation of basement membrane and dermal collagen matrix^60,63^.

In the absence of vitamin C, the synthesis of fibroblast cells and crosslinking decreased according to many studies^54,58,64–67^, showing the dependence of the collagen hydroxylase enzymes on vitamin C. Hydroxylation by vitamin C stabilizes the collagen molecule. It stimulates collagen mRNA production by fibroblasts^63,68^. In addition to vitamin C’s ability to promote collagen synthesis^58,64^, evidence suggests that vitamin C increases proliferation and migration of dermal fibroblasts^63,67,69^, functions vital for effective wound healing. However, the underlying mechanisms driving this activity are unknown, and vitamin C has been shown to increase the repair of oxidatively damaged bases^63^. As a water-soluble and charged molecule, vitamin C is repelled by the physical barrier of the terminally differentiated epidermal cells. Only when pH levels are below four and vitamin C does some penetration occur^70^. Also, the physical effects of plasma revealed the gradual reduction in pH by increasing the treatment time of air plasma^71^. The pH depends on primary free radicals generated by plasma and secondary free radicals produced by interaction with the skin. The pH decreasing could be related to the positive ions, e.g., H_2_O^+^, as was suggested previously by Tochikubo et al.^72^, or because of a higher concentration of NO_X_ species in the discharge volume^73^. Therefore plasma treatment could increase vitamin C absorbance by increasing the wettability of skin and decreasing the PH. High levels of ROS can cause tissue injury and aging^74^. On the other hand, antioxidants can scavenge ROS^74^. So, using vitamin C as an antioxidant can quench excess ROS and reduce the formation of damaging ROS^74–76^, produced by the cold atmospheric plasma.

As said before, collagen is one of the most important structural proteins of the skin. The presence of this protein in the skin causes endurance, firmness, and elasticity of the skin^1^. With age, collagen production will decline^2,3^. This decrease in collagen production in the skin can make the skin vulnerable, resulting in increased wrinkles and at the same time reduced skin smoothness and softness^1,4,5^. Therefore, if we can improve collagen production in the skin by the desired method, we can positively affect skin rejuvenation. Thus, the average thickness of collagen in the samples of skin was examined.

The collagen is synthesized by fibroblast cells^7^. Collagen is a triple helix composed of three polypeptides^77^ which have thermally unstable and chemically stable bonds. When the collagen forming tissues are heated, this protein matrix’s physical properties change at a specific temperature, which is observed as a shrinkage, causing the collagen to change. Soft tissue remodeling is a biophysical phenomenon that occurs on a cellular and molecular scale. Molecular contraction or partial denaturation of collagen is energy-dependent, destabilizing the molecule’s longitudinal axis by cleaving the unstable ternary helical heat bonds. As a result, stress is created that breaks the matrix’s intermolecular bonds, which is a quick extracellular process. At the same time, the cellular shrinkage, which is usually required for cell proliferation, takes longer^78^.

Fibroblast cells proliferate at the processing site 72 h after heat treatment. These proliferating cells become contractile myofibroblast cells that are the source of soft tissue shrinkage. After the cellular shrinkage, collagen is stretched into soft tissue. This restructuring can alter the consistency and geometry of the soft tissue and be used for aesthetic purposes^78^.

All groups except the high-voltage plasma group with vitamin c showed a slight decrease in the mean collagen area after 4 weeks of plasma processing related to the collagen shrinkage. Also, the different trend of high-voltage plasma group with vitamin c can be due to both methods, which caused this process to occur before the first punch and the skin reaction to be rapid.

In all three groups that received plasma treatment, the mean collagen level of the biopsy specimen was significantly increased 6 weeks after the plasma process compared to the control group. This significant increase in the mean collagen level indicates an increase in collagen protein synthesis after plasma treatment. One of the reasons is applying immediate and controlled thermal effects. On the other hand, the presence of NO species in the plasma also increased fibroblasts, resulting in collagen synthesis. This new collagen production is one of the most critical factors in skin regeneration and rejuvenation.

The outermost layer of the skin is the epidermis, which comprises various layers. The deepest layer of the epidermis is the basal layer, which is the skin cells’ birthplace. The epidermis protects the human body against external factors, such as heat and cold. The epidermis thickness varies in different body areas; for example, the epidermis has the lowest thickness in the eyelids and is the thickest in the palms^79,80^. In this study, the thickness of the epidermis layer was evaluated in the fourth and sixth weeks after plasma processing.

As shown in the results of the epidermal layer thickness, an increase in the epidermal layer is observed 4 weeks after processing due to the protective role of the epidermis layer against heat. However, the thickening trend decreases by the sixth week and slows down. In the high-voltage plasma treatment with vitamin C ointment, this thickness decreased. This reduction is minor, results are higher than the control, and the skin has a suitable thickness. In this specimen, the process of collagen growth was relatively upward, and the collagen layer was highly thick because of the limited space of the skin; maybe this high thickness prevented the thickening of the epidermal layer. As can be seen, the results of physical (elastic) testing confirm this data.

In the end, many other research groups worked on plasma skin rejuvenation with complex and costly devices along with complex equipment, which is difficult and impossible to be used at homes or beauty salons. So, in this paper, a device was used that was effective, easy to use, and inexpensive, and according to the presented results, it can be used at homes and beauty salons. Also, this study showed that as a new method, vitamin C ointment could be used along with cold plasma to improve the results of plasma skin rejuvenation.

## Conclusions

Plasma skin rejuvenation is one of the new techniques that can stimulate skin rejuvenation with a short recovery period and without significant side effects and tissue damages. The portable PSR device designed in this research is easy to use, and this study showed that a portable plasma device could have a good effect on skin rejuvenation. It can promote the mechanical parameters (maximum stress, work-up to maximum force, and elastic stiffness) due to improved histological parameters, such as the average collagen and epidermal thickness. Also, the use of vitamin C ointment can improve the results and accelerate the rejuvenation process.

## Materials and methods

### Ethical considerations

In the present study, male Wistar rats weighing 250 ± 50 g were used in separate cages. There were five rats in each of the four groups. They were kept under standard laboratory conditions (room temperature, atmospheric pressure, 30 ± 10 humidity, 12-h day–night cycle) with easy access to water and food. The Medical Ethics Committee approved all animal experiments of Shahid Beheshti University of Medical Sciences (IR.SBMU.RETECH.REC, ethic no. 1396.28). All surgeries were performed under deep anesthesia under the guidance of Shahid Beheshti University of Medical Sciences, and the maximum effort was made to minimize animal abuse. For sacrificing rodents, we chose inhalation methods with CO_2_. To make this method more ethical, the animals were fully unconscious (anesthetized with the same method explained in the article) before putting them into the CO_2_ chamber. The CO_2_ gas used for this study was 99% (more than 70%) and entered the chamber directly. Only one rat was put into the chamber each time and removed after the death was confirmed. All methods were carried out under the relevant guidelines and regulations, and this study was carried out in compliance with the ARRIVE guidelines.

### Portable plasma device

The floating electrode dielectric barrier discharge (FEDBD-Plasma Fanavar Jam Company) was used to generate cold plasma in this study, and it is shown schematically in Fig. 1b, which explains different parts of the device. The approximate dimensions of the device were 5 cm × 5 cm × 20 cm. Peak-to-peak voltages for low- and high-voltage modes were 5.2 kV, and 7.2 kV, respectively, and a frequency of 20 kHz was applied in both modes. An oscilloscope (Tektronix, DPO3012), a high-voltage probe (Tektronix, P6015A, 1:1000), and a current probe (TCP-202) were used for the investigation of the electrical characteristics. The effects of these two radiation modes and the application of vitamin C ointment are then investigated on the rats' skin.

### Optical emission spectroscopy

Different active species of cold plasma were studied by optical emission spectrometry (OES; Avaspec‐3648‐USB2), and a 600 μm optical fiber cable (FC‐UV600‐2‐SR) was connected to record the spectral emissions.

### Preparation and treatment

To investigate the effect of portable plasma on skin rejuvenation, four groups of five Wistar rats with a mean age of approximately 4 months were considered. The rats were divided into fourth treatment groups; the first group just received vitamin C ointment. Low voltage plasma for the second group and high voltage for the third group were used, and both received vitamin C ointment. In the fourth group, a high voltage of the device without ointment was used. On the back of the rats, two areas of about 30 cm^2^ were identified. The hair on that surface was trimmed using scissors. Processing time in this area was 5 min, and the skin surface of the rat was cleaned. The processing times were performed on every other day basis (3 sessions per week).

For plasma irradiation, the rats were anesthetized by injecting ketamine hydrochloride (100 mg/kg) and xylazine hydrochloride (10 mg/kg). Lidocaine was also used for local anesthesia just before punching. After anesthetizing the rats, their dorsal hair (near the neck and behind the chest) was shaved, and the target area was marked and treated with a portable plasma device (Fig. 1d).

A punch with a 6-mm diameter was used for a skin biopsy. There were two areas on the back of the rats: one did not receive plasma (untreated area), and the second received plasma (treated area). The distance between these two areas was 1.5 cm. the skin samples (6 mm punch biopsies) were taken from the control area (untreated area) right before treatment. Twelve sessions after plasma processing, the second skin biopsy was taken right after treatment on session 12. The specimen was sutured after sampling. Also, right after treatment on session 18, rats were sampled from the treated area for the third time. After the third sampling, the plasma processing was stopped. Samples were stored in 10% formalin stabilizing solution.

### Thermal evaluation

Plasma temperature is an important parameter in medical applications, and its thermal effect on the living tissue must be investigated in terms of safety. To do so, the normal skin temperature of the rat during 5 min of plasma irradiation with low and high voltages was monitored by a thermal camera (FLIR-E63900-T198547, Estonia, thermal sensitivity < 0.06 °C).

### Mechanical skin test (stretch)

After sacrificing the rats, we stripped the skin to 60*40 mm to prepare the specimens. The samples were kept separately moistened with 0.9% NaCl solution at − 20 °C until testing. In this test, the skin strips were placed between the clamps of the Zwick-Roll, Z25-ph1F, Germany device, and the effect of uniaxial tensile at a constant speed of 10 mm/min until break-in was evaluated. Mechanical parameters (maximum stress (N/mm^2^), work-up to maximum force [W up to F_max_ (Nmm)], and elastic stiffness [E-Modulus (N/mm^2^)] were calculated by a computer connected to the test tools. The mechanical results were assessed to evaluate the effect of plasma on the mechanical tissue parameters. The maximum tolerance calculates the maximum force (N) that a material (here skin) can withstand before rupturing under tension, pressure, etc. The maximum stress (N/mm^2^) is obtained by dividing the maximum force over the specimen’s cross-sectional area. The amount of work-up to the maximum force [W up to Fmax (Nmm)] is obtained by calculating the area under the curve and expressing the energy absorbed by the tissue under the applied tensile force. The elastic stiffness [E-Modulus (N/mm^2^)] of an object is defined as the linear part of the stress–strain curve slope.

### Histological analysis

After easy death using CO_2_ inhalation, rats were sampled, as mentioned previously, at sessions 12 and 18, and the samples were fixed in a 10% formalin stabilizing solution. The samples were dried in alcohol series, then cleaned with xylene and molded-in paraffin to be prepared for H&E (hematoxylin–eosin) staining and Masson’s Trichrome specific staining. Stained tissues were then imaged with a Kf2 optical microscope (ZEISS West Germany^®^) equipped with a scaled lens and a scaled cuticle (Erma Japan^®^) with 10 objective lens magnification and a Sony Cybershot camera (4 × 10 × 40 magnification).

The average collagen area is essential because of the increase in the number of collagens, which is one of the factors affecting skin rejuvenation. ImageJ and Carl Zeiss Axiovision Rel. 4.8. The software was used to do measurements and calculate the collagen density, and the area of collagen was determined.

### Statistical analysis

Results were expressed as mean ± standard error of the mean (mean ± SEM). Statistical analysis of data was performed by applying One way-ANOVA to compare the groups using the Graph Pad Prism (9.0.0) software. The significance level was considered less than 0.05 (p < 0.05).

### Ethical approval

The proposal of study was approved by the Ethics Committee, deputy of research, Shahid Beheshti University of Medical Sciences, Tehran.
